# Analysis of the diagnostic accuracy of rapid antigenic tests for detection of SARS-CoV-2 in hospital outbreak situation

**DOI:** 10.1007/s10096-021-04346-8

**Published:** 2021-11-18

**Authors:** Jesús María Aranaz-Andrés, Abelardo Claudio Fernández Chávez, Amaranta McGee Laso, Melanie Abreu, Paloma Moreno Núñez, Juan Carlos Galán, Rafael Cantón Moreno

**Affiliations:** 1 Preventive Medicine and Public Health, Hospital Universitario Ramón Y Cajal and Instituto Ramón Y Cajal de Investigación Sanitaria (IRYCIS), Madrid, Spain; 2 CIBER Epidemiología Y Salud Pública (CIBERESP), Instituto de Salud Carlos III, Madrid, Spain; 3 Universidad Internacional de La Rioja (UNIR), Logroño, Spain; 4 Servicio de Microbiología, Hospital Universitario Ramón Y Cajal and Instituto Ramón Y Cajal de Investigación Sanitaria (IRYCIS), 28034 Madrid, Spain; 5 Red Española de Investigación en Patología Infecciosa (REIPI), Instituto de Salud Carlos III, Madrid, Spain

**Keywords:** COVID-19, Asymptomatic screening, Rapid antigen tests

## Abstract

The reverse transcriptase polymerase chain reaction (RT-PCR) continues to be the reference diagnostic method for the confirmation of COVID-19 cases; however, rapid antigen detection tests (RADT) have recently been developed. The purpose of the study is to assess the performance of rapid antigen-based COVID-19 testing in the context of hospital outbreaks. This was an observational, cross-sectional study. The study period was from October 2020 to January 2021. The “Panbio COVID-19 AG” RADT (Abbott) was performed and TaqPath COVID-19 test RT-PCR. The samples were obtained from hospitalised patients in suspected outbreak situations at the Ramón y Cajal Hospital. A hospital outbreak was defined as the presence of 3 or more epidemiologically linked cases. The sensitivity, specificity, positive predictive value (PPV), and negative predictive value (NPV) of the RADT were calculated using RT-PCR as a reference. A total of 17 hospital outbreaks were detected in 11 hospital units during the study period, in which 34 RT-PCR and RADT screenings were performed. We obtained 541 samples, which were analysed with RT-PCR and a further 541 analysed with RADT. Six RADT tests gave conflicting results with the RT-PCR, 5 of them with a negative RADT and positive RT-PCR and one with positive RADT and a negative RT-PCR. The sensitivity of the RADT was 83.3% (65.3–94.4%) and the specificity was 99.8% (98.9–100%). The PPV was 96.2% (80.4–99.9%) and the NPV was 99% (97.7–99.7%). The RADT shows good diagnostic performance in patients on non-COVID-19 hospital wards, in the context of an outbreak.

## Introduction

In the current COVID-19 pandemic period, outbreaks have occurred at both community and hospital levels [1].

The presence of three or more epidemiologically linked cases is defined as a hospital outbreak of SARS-CoV-2 and an outbreak is considered over when there are no more epidemiologically linked cases [2]. A group of cases that have no epidemiological link is defined as a cluster [3]. In addition, selective screening without an outbreak is defined as testing performed on hospital wards with an apparently higher risk of viral transmission or where clusters of cases have appeared.

The basic strategy for breaking the transmission chains of SARS-CoV-2 in the above contexts is the follow-up and diagnosis of the patients involved. The diagnostic technique of choice for confirmation of COVID-19 in any context is reverse transcriptase polymerase chain reaction (RT-PCR), but other less technically complex diagnostic tools such as rapid antigen detection tests (RADT) have been developed, which have contributed to the generalisation of viral detection tests. Although somewhat less sensitive and specific than RT-PCR, these tests are faster (they give results in 15 min) and cost-effective, which has allowed the World Health Organization (WHO) to recommend their use under certain circumstances, such as close contact and suspected cases with a symptomatic evolution of less than 5 days [4].

However, there is no consensus that RADT cannot be used in other contexts. RADT testing has been authorised for use in asymptomatic persons in numerous countries [5], while the CDC, in a set of guidelines for the use of RADT in different fields, has endorsed its use for serial testing in closed populations [5].

In Europe, the European Commission has facilitated the use of RADT, ensuring access by the Member States [6]. In its latest technical documents, the ECDC states that RADT can help reduce transmission by detecting highly infectious cases early, to allow contact tracing to start quickly [7]. In Spain, specifically in Madrid, RADT has been included in COVID-19 early detection, surveillance, and control strategies [2] [8] [9].

Despite the above, there is little scientific evidence of its efficacy in hospital outbreaks. On the other hand, there are studies that have been carried out applying other diagnostic techniques such as computed tomography in the detection of asymptomatic patients with COVID-19 [10].

The purpose of the study is to assess the performance of rapid antigen-based COVID-19 testing in the context of hospital outbreaks as the method of choice.

## Methods

This cross-sectional study was carried out from October 2020 to February 2021 in the hospitalisation units of the Ramón y Cajal Hospital aimed at accommodating patients with conditions other than COVID-19 where there were hospital outbreaks of SARS-CoV-2 infection. Once an outbreak was declared, additional hospital admissions were prohibited in the affected unit. The centre is a highly complex hospital with approximately 900 beds. Most of the patients are hospitalised in double rooms. The study was approved by the Hospital Ethics Committee (reference 356/20).

Two types of tests were used to diagnose and follow-up patients involved in each outbreak: [1] The “Panbio COVID-19 AG Test” antigen test (Abbott Chicago, MI), available at Ramón y Cajal University Hospital since October 2020, was used. The result is displayed on a chromatographic, immunoassay-based platform [11]. The test was considered positive as indicated in the data sheet. According to this, the clinical performance data of the Panbio COVID-19 AG Test calculated using an FDA US RT-PCR reference showing an analytical sensitivity of 91.1% (95% CI: 84.2–95.6%) and a specificity with 99.7% power (95% CI: 98.6–100.0%). [2] The other test used was RT-PCR that was performed using the TaqPath COVID-19 CE-IVD RT-PCR Kit manufactured by Thermo Fisher Scientific (Waltham, MA, USA) that targets orf1ab, N, and S genes. A positive result was defined when at least two of the three SARS-CoV-2 targets were detected.

The cycle threshold (Ct) of all positive PCR was obtained in RT-PCR and its value was compared with the result obtained in the associated RADT. Only samples with Ct value ≤ 35 were considered positives.

All RT-PCR and RADT tests administered to patients over 18 years of age, hospitalised in units affected by an outbreak, and without COVID-19 compatible symptoms were included.

Tests administered to patients under 18 years of age, unconscious, with mental disorders, with inability to understand written or spoken language, or with documented records of having had SARS-CoV-2 infection in the preceding months were excluded. Tests administered to the patient with an interval of more than 48 h between RT-PCR and RADT were also excluded.

When an outbreak was detected by the presence of three or more epidemiologically linked cases confirmed with RT-PCR, screening commenced for all patients admitted to that area. Every patient in the unit was managed equally, with isolation precautions and periodic screening. Two nasopharyngeal exudate samples were obtained from each patient with an interval of no more than 48 h for diagnostic testing. Diagnostic tests were not always performed in the same order, and in some cases, the staff taking the second sample were aware of the result obtained in the first sample. The main reason why there is a delay between tests in 8.4% of our sample is because the staff instructed in the use of RADT was not available at that moment, and the performance of RADT was delayed. When the PCR was negative, regardless of the outcome of the RADT, the patient was re-screened every 5 days and until the outbreak was considered over. Only when PCR was positive for SARS-CoV-2, regardless of the outcome of the RADT, was the patient transferred to a COVID-19 unit and follow-up ended.

To improve the consistency of the sampling method, the procedure was always carried out by the same nursing team from the Preventive Medicine Service.

The minimum sample size required was calculated using the Wald test, considering a study power of 80%, a significance level of 0.05, a zero-sensitivity ratio (p0) of 0.85, and an alternative sensitivity ratio (pa) of 0.80. The sample size determined was 503 samples per technique.

### Study variables and sources of information

1. Sociodemographic variables: age, sex, and risk factors were obtained from the electronic medical record (EMR). We used the risk factors for COVID-19 selected by the CDC as having a poor prognosis of the disease with a reliable and mixed degree of evidence [12]. According to the CDC, adults of any age with the following risk factors are at increased risk of becoming seriously ill with COVID-19: cancer, chronic kidney disease, COPD, Down syndrome, cardiovascular diseases (cerebrovascular, coronary heart disease, heart failure, cardiomyopathy), immunosuppression due to organ transplantation and/or immunosuppressant, obesity, pregnancy, sickle cell disease, and type 2 diabetes.
2. Microbiological variables: date of test and RT-PCR result, including the cycle threshold, were provided by the Microbiology Department.
3. Microbiological variables: test administration and RADT results, which were obtained from the Preventive Medicine database.

### Statistical analysis

The sensitivity, specificity, positive predictive value (PPV), and negative predictive value (NPV) of the RADT test were calculated using RT-PCR as a reference. The analyses were calculated with a confidence interval of 95% (95% CI).

## Results

There were 17 hospital outbreaks detected in 11 hospital units during the study period. A total of 34 RT-PCR and RADT screening rounds were performed. We obtained 541 samples, which were analysed with RT-PCR and a further 541 analysed with RADT. In our study, 91.57% (495) of the cases were screened simultaneously. In only 8.4% (46), it was done with a separation between 12 and 48 h. The number of patients studied was 371. Most screening took place in the gastroenterology unit with 9, followed by Internal Medicine, with 5. Only one screening was required in the Oncology, Vascular Surgery, and General and Digestive Surgery units.

Table 1 shows the spatial and temporal distribution of the screenings and their cluster by outbreaks. It also summarises the results of the 541 samples studied. A total of 25 (4.6%) of RADT performed were true positives, with reference values of the results of the RT-PCR. Likewise, 510 (94.2%) of RADT were true negatives.

**Table 1:** Results of RADT performed in the context of the outbreak, taking the results of RT-PCR as reference

Hospitalisation units	Screened by outbreak	Outbreaks	RADT + RT-PCR +	RADT − RT-PCR −	RADT − PCR +	RADT + PCR −	% positive
Oncology	1	Outbreak 1	0	26	0	0	0.0%
General Surgery	1	Outbreak 1	0	23	0	0	0.0%
General Surgery	2		0	18	0	0	0.0%
General Surgery	1	Outbreak 2	0	23	0	0	0.0%
Trauma Service	1	Outbreak 1	0	13	0	0	0.0%
Trauma Service	2		0	6	0	0	0.0%
Trauma Service	1	Outbreak 2	2	19	0	1	9.1%
Trauma Service	2		3	6	1	0	30.0%
Internal Medicine	1	Outbreak 1	1	14	1	0	6.3%
Internal Medicine	2		1	4	0	0	20.0%
Internal Medicine	3		0	3	0	0	0.0%
Internal Medicine	1	Outbreak 2	0	22	0	0	0.0%
Internal Medicine	2		0	19	0	0	0.0%
Cardiology	1	Outbreak 1	2	16	0	0	11.1%
Cardiology	2		3	3	0	0	50.0%
Neurology	1	Outbreak 1	0	18	2	0	0.0%
Neurology	1	Outbreak 2	0	12	0	0	0.0%
Neurology	1	Outbreak 3	0	16	0	0	0.0%
Gastroenterology	1	Outbreak 1	0	34	0	0	0.0%
Gastroenterology	2		3	17	0	0	15.0%
Gastroenterology	3		0	14	0	0	0.0%
Gastroenterology	4		0	10	0	0	0.0%
Gastroenterology	1	Outbreak 2	0	24	0	0	0.0%
Gastroenterology	1	Outbreak 3	1	26	0	0	3.7%
Gastroenterology	2		1	31	1	0	3.0%
Gastroenterology	3		0	5	0	0	0.0%
Gastroenterology	4		2	8	0	0	20.0%
Respiratory Unit	1	Outbreak 1	1	18	0	0	5.3%
Respiratory Unit	2		1	7	0	0	12.5%
Respiratory Unit	3		0	3	0	0	0.0%
Vascular Surgery	1	Outbreak 1	0	13	0	0	0.0%
Infectious Diseases	1	Outbreak 1	1	13	0	0	7.1%
Infectious Diseases	2		1	14	0	0	6.7%
Infection Diseases	3		2	12	0	0	14.3%
	34	17	25	510	5	1	4.6%

*RADT*, rapid antigen tests; *PCR*, polymerase chain reaction

A total of 60% patients with positive RADT had undergone two screenings before being diagnosed with COVID-19, while 31.2% and 16.5% of patients with RADT and PCR negative had undergone two and three screening tests, respectively, during hospital outbreak follow-ups (Table 2). The mean duration of RADT positivity from the outbreak declaration was 3 days.

**Table 2:** Number of screenings performed per patient

	Total, of patients *n* (%)	RADT + PCR + *n* (%)	RADT − PCR − *n* (%)	RADT − PCR + *n* (%)
Number of patients in single screening	192 (51.7%)	10 (40.0%)	178 (52.3%)	3 (66.7%)
Number of patients undergoing two serial screenings	123 (33.2%)	15 (60.0%)	106 (31.2%)	2 (33.3%)
Number of patients undergoing three serial screenings	56 (15.1%)	0 (0.0%)	56 (16.5%)	0 (0.0%)

*RADT*, rapid antigen tests; *PCR*, polymerase chain reaction

In total, there were six RADT that yielded opposite RT-PCR results, five of them with negative RADT and positive RT-PCR and one with positive RADT and negative RT-PCR. One of these samples with discorded results (negative RADT and positive RT-PCR) was obtained with an interval between 24 and 48 h; the remaining pairs of conflicting samples were taken on the same day. With these data, RADT sensitivity was 83.3% and specificity was 99.8% (Table 2). With a prevalence of COVID-19—a percentage of positives in the sample—of about 5%, the positive predictive value (PPV) was 96.2% and the negative predictive value (NPV) was 99.0%.

**Fig. 1:**
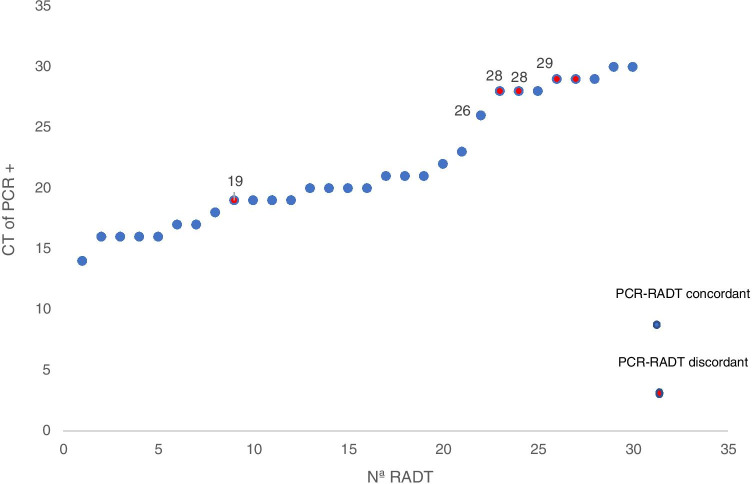
Ct of positive PCR (*n* = 30) and the result of its associated RADT

Four of the five cases with RADT − /PCR + had Ct values between 25 and 30 (Fig. 1), reinforcing their consideration as false negatives. The Ct value of the only positive RADT and negative PCR was not recoded but was higher than 35.

When using RT-PCR as gold standard for SARS-CoV-2 detection, the agreement between RADT and RT-PCR was 95.4% when Ct < 25.

More than 56% of all participating patients were men. The percentage of men in the negative RADT group (negative RT-PCR) was 58.5% and in the positive RADT group (RT-PCR negative was 36%).

Median age was higher (83.7 years) in patients with a positive RADT and positive PCR compared with those with a negative RADT and PCR (75.8 years).

According to clinical characteristics of the included patients, Hypertension (HTN), chronic lung disease, and cardiovascular disease were the most common risk factors among patients with positive RADT and RT-PCR. To a lesser extent, HTN and cardiovascular disease were the most prevalent among patients with negative RADT and RT-PCR. The most common risk factor among the five patients with negative RADT and PCR positive was cardiovascular disease (Table 3).

**Table 3:** Analysis of the diagnostic validity of RADT in the context of hospital outbreak by COVID-19. Reference technique: RT-PCR

Total RADT	**541**
*Prevalence of COVID +	4.6%
Sensitivity	83.3% (95% CI: 65.3%, 94.4%)
Specificity	99.8% (95% CI: 98.9%, 100%)
PPV	96.2% (95% CI: 80.4%, 99.9%)
NPV	99.0% (95% CI: 97.7%, 99.7%)

*PPV*, positive predictive value; *NPV*, negative predictive value; *RADT*, rapid antigen tests. *Percentage of positive patients in the sample

Patients with positive RADT and RT-PCR had more risk factors than patients with negative RADT and RT-PCR (Table 4).

**Table 4:** Clinical characteristics of the patients included in the study according to the results of the diagnostic tests (RADT, RT-PCR)

	Total patients *n* (%)	RADT + PCR + *n* (%)	RADT − PCR − *n* (%)	RADT − PCR + *n* (%)
Total	**371 (100%)**	**25 (6.7%)**	**340 (91.6%)**	**5 (0.01%)**
Clinical features				
Age (median)	76.7	83.7	75.8	85.9
Men	208 (56%)	9 (36%)	199 (58.5%)	1 (20%)
HTN	226 (60.9%)	19 (76.0%)	204 (60.0%)	3 (60.0%)
Cancer	99 (26.7%)	10 (40%)	88 (25.9%)	1 (20.0%)
Cerebrovascular disease	44 (11.9%)	4 (16.0%)	39 (11.5%)	1 (20.0%)
Chronic kidney disease	42 (11.3%)	4 (16.0%)	37 (10.9%)	1 (20.0%)
Chronic lung disease	50 (13.5%)	5 (20.0%)	43 (12.7%)	2 (40.0%)
Cardiovascular disease	129 (34.8%)	11 (44.0%)	114 (33.5%)	4 (80.0%)
Obesity	20 (5.4%)	3 (12.0%)	17 (5.0%)	0 (0.0%)
Immunosuppressive therapy	1 (0.3%)	0 (0.0%)	1 (0.3%)	0 (0.0%)
Solid organ transplantation	18 (4.9%)	0 (0.0%)	18 (5.3%)	0 (0.0%)
Type 2 diabetes	95 (25.6%)	8 (32%)	86 (25.3%)	1 (20.0%)
Patients without RF	73 (19.7%)	5 (20.0%)	66 (19.4%)	1 (20.0%)
Patients with 1 RF	74 (20.0%)	3 (12.0%)	71 (20.9%)	0 (0.0%)
Patients with 2 RF	95 (25.6%)	2 (8.0%)	92 (27.1%)	1 (20.0%)
Patients with three or more RF	129 (34.8%)	15 (60.0%)	111 (32.65%)	3 (60.0%)

*RADT*, rapid antigen tests; *PCR*, polymerase chain reaction; *HTN*, hypertension; *RF*, risk factor

## Discussion

According to the present study, rapid SARS-CoV-2 antigen detection tests have shown high specificity and sensitivity. Therefore, it is reasonable to consider due to their lower price and the speed with which they can provide results (15 min), they can be used in the context of hospital outbreaks, even when PCR is available. This fact is in line with CDC recommendations in December 2020, which states that serial RADT testing for SARS-CoV-2 is reasonable for fast diagnosis and preventing transmission [5]. The CDC does not foresee the need for tests to confirm the negative results of a RADT test. The frequency of repeat testing in the population while the outbreak is active is yet to be determined.

In our model, the sensitivity of RADT compared to RT-PCR was globally very good (83.3%), among asymptomatic population in the moment of screening. However, five patients would have been unidentified only using RADT strategy and RT-PCR would have lost 2 patients with only one screening. These data suggest that in an outbreak situation, we must repeat the screening periodically.

Although RT-PCR could yield more sensitive data, it is a time-consuming strategy, which might be a handicap in an outbreak situation as is described in this work.

However, both the data sheet of the test used (Panbio COVID-19 AG) and some studies indicate that a negative RADT test is not sufficient to rule out SARS-CoV-2 infection. Unlike the systematic review carried out by Deeks, which assessed the diagnostic performance of RADT depending on the patient’s symptoms and when the diagnostic tests were performed, our study focused on patients hospitalised in a non-COVID-19 unit where a COVID-19 outbreak was declared. However, both studies agree on the results of PPV when the prevalence of COVID-19 was 5% [13]. Ian W. Pray, in an evaluation of the performance of RADT Sofia (FIA) compared with RT-PCR, concludes that negative RADT results in symptomatic persons and RADT positive results in asymptomatic persons should be confirmed by molecular testing [14]. Although no validation of sensitivity and specificity of RADT has been performed in the context of hospital outbreaks, several researchers have done so in asymptomatic populations without an epidemiological history of interest. For example, Turcato et al. [15] analysed the validity of the Standard Q COVID-19 AG SD biosensor in a sample of 2419 subjects attending the emergency department for a non-COVID-19 condition and obtained a sensitivity of 50.0% (95% CI: 36.0–63.0%) and a specificity of 99.6% (95% CI: 99.1–99.9%). Another study published by Okoye et al. [16] obtained similar results among 2645 asymptomatic university students with the Abbott BinaxNOW COVID-19 Antigen Card test. The variation between ad hoc estimates of sensitivity presented by some papers and others is striking. Beyond the variability inherent in random error, the selection of samples with a low pre-test probability (general population, asymptomatic subjects) suggests that the confidence intervals calculated for sensitivity are wider than those obtained for specificity. This is also evident in our results. Although sensitivity and specificity are strictly applicable to the diagnostic test and should not be altered by the prevalence of the disease, it is considered appropriate to check the validity of a diagnostic test in populations of different characteristics, since variability in the severity spectrum of the disease is an element that determines these parameters in test validation studies.

To note that our study was conducted in asymptomatic patients involved in a hospital outbreak. This use apparently contradicts that recommended in the RADT data sheet—patients with less than 5 days of symptoms [11]—but it must be taken into account that the diagnosis of a subject is not comparable to a serial diagnostic procedure, with repeated tests, which is the hospital’s usual course of action when analysing an outbreak [5]. A total 60% of patients with a positive RADT obtained this diagnosis at the second screening and not at the first screening after the outbreak was declared.

Our results are in agreement with previous works, where RADT tests were compared with RT-PCR, attending to Ct values. When Ct value was lower than 25, the sensitivity was 99.5% among symptomatic patients [17]; however, among asymptomatic patients, the sensitivity was 95.8%, which is very close to 95.4% observed in our result when Ct ≤ 25. However, when Ct > 25, the sensitivity drops drastically [18]. In a valid diagnostic study, it would have been interesting to know the exact Ct value of the only case where there was a contradiction between the PCR − and RADT + that we had in the study; however, this was higher than 35 and considered negative per protocol interpretation. We do know that 72 h after performing the RADT, the patient obtained a PCR + with a Ct of 18. This patient’s age and risk factors were no different from those of the other patients with positive RADT results. This finding is most likely coincidental; the hypothesis that, on certain occasions, RADT may detect cases before PCR does not appear to be biologically or technically plausible and may be related to sampling.

In our study, we obtained high NPV values (99.0%, 95% CI: 97.7%, 99.7%) with a prevalence of 4.6% positive patients consistent with RT-PCR. Given the NPV value, with a prevalence equal to or greater than that mentioned in the non-COVID hospital setting, screening in outbreaks with RADT without confirmation PCR could be established.

The distribution of age and risk factors in sick and healthy patients is consistent with evidence accumulated so far [12]. The prevalence of comorbidities in hospitalised patients is high; therefore, in the hospital setting, it is particularly important to have a rapid, effective, and efficient diagnostic test to detect cases and avoid nosocomial transmission.

Although the data provided by this paper suggests that this is feasible, it would be advisable to quantify this more accurately in subsequent research and in the same context that of the hospital outbreak to determine how many cases go undiagnosed when replacing PCR with RADT and how many infections can be avoided by speeding up the diagnosis by replacing one test with another. Even if an unfavourable result is obtained, it may be that, in certain hospitals and depending on the extent of the difference, economic savings and, above all, the early redistribution of patients and the consequent reorganisation of the affected hospital stay units may make it advisable to choose RADT instead of the PCR. All these decisions will have to be made considering the expected prevalence at each moment.

It is important to note that despite having fixed a time interval between pairs of nasopharyngeal exudates of maximum 48 h, only 8.3% of sample pairs were taken more than 24 h apart.

Finally, one of the limitations we have found is that, on very rare occasions, the staff taking the samples knew the result of one of them (usually PCR) that had been administered prior to diagnostic screening and this could have conditioned the way the remaining test sample was taken (usually RADT), introducing a bias that would improve the concurrence of the test results.

## Conclusions

RADT show good diagnostic performance in patients in the context of an outbreak of COVID-19 in hospital units occupied by non-COVID-19 patients in a high-complexity hospital in Madrid. Although the data sheet does not recommend its use in asymptomatic patients, the impact of RT-PCR replacement with RADT on diagnostic sensitivity in serial detection strategies in closed healthcare settings should be quantified. Once this has been done, the diagnostic strategy could be adapted to the epidemiological characteristics and economic and logistical possibilities of each hospital.

## Data Availability

Yes. The datasets generated during and/or analysed during the current study are available from the corresponding author on reasonable request.

## Code availability

Yes.
